# TPI1 promotes malignant progression and ferroptosis resistance in head and neck squamous cell carcinoma

**DOI:** 10.3389/fgene.2026.1889940

**Published:** 2026-08-12

**Authors:** Rui Ye, Zhihua Xu, Yehai Liu

**Affiliations:** 1 Department of Otorhinolaryngology Head & Neck Surgery, The First Affiliated Hospital of Anhui Medical University, Hefei, China; 2 Department of Otorhinolaryngology, The Third Affiliated Hospital of Anhui Medical University (The First People’s Hospital of Hefei), Hefei, China

**Keywords:** ferroptosis, HNSCC, prognostic biomarker, TPI1, tumor progression

## Abstract

**Objective:**

Triosephosphate isomerase 1 (TPI1) is aberrantly overexpressed and exerts a critical oncogenic role in the development and progression of various human cancers by regulating multiple malignant biological phenotypes. However, the biological functions of TPI1 and the molecular mechanisms underlying its regulatory effect on ferroptosis sensitivity in head and neck squamous cell carcinoma (HNSCC) remain not fully elucidated.

**Methods:**

TPI1 expression was analyzed across 33 cancer types using The Cancer Genome Atlas pan-cancer dataset, and validated in 6 paired HNSCC tumor/adjacent normal tissues via Western blotting and immunohistochemistry. Clinical correlations were assessed using the Wilcoxon rank-sum test, and prognostic value was evaluated by Kaplan-Meier analysis and multivariate Cox regression. A prognostic nomogram integrating TPI1 and clinical covariates was constructed and validated by calibration curves. Single-cell RNA-sequencing (3 independent cohorts: GSE103322, GSE150321, GSE172577) and spatial transcriptomics (4 patient samples) were employed to map TPI1 cellular distribution in the tumor microenvironment. Gain- and loss-of-function assays were performed in FaDu (overexpression) and TU177 (knockdown) cell lines using lentiviral vectors. Cell proliferation was measured by CCK-8 and colony formation assays; migration and invasion were evaluated by wound healing and Transwell assays. Ferroptosis sensitivity was assessed by RSL3 dose-response curves, malondialdehyde (MDA) assay, BODIPY 581/591 C^11^ lipid peroxidation staining, and intracellular Fe^2+^ quantification.

**Results:**

TPI1 was significantly upregulated in HNSCC at both mRNA and protein levels, with an AUC of 0.914 for distinguishing tumor from normal tissues. High TPI1 expression correlated with male sex, high histologic grade, advanced pathologic stage, and T3-T4 stage, and independently predicted poor overall survival. The nomogram integrating TPI1, age, gender, stage, and grade showed robust prognostic performance with well-calibrated 1-, 3-, and 5-year survival predictions. Single-cell and spatial transcriptomics confirmed that TPI1 was predominantly and specifically expressed in malignant epithelial cells, with minimal expression in stromal and immune cells. Functionally, TPI1 overexpression significantly enhanced FaDu cell proliferation, migration, and invasion, while TPI1 knockdown markedly suppressed these phenotypes in TU177 cells. Mechanistically, TPI1 conferred ferroptosis resistance by attenuating RSL3-induced lipid peroxidation, reactive oxygen species accumulation, and intracellular Fe^2+^ elevation; conversely, TPI1 silencing sensitized HNSCC cells to ferroptotic cell death.

**Conclusion:**

This study identifies TPI1 as a key tumor-promoting factor and independent prognostic biomarker in HNSCC. TPI1 promotes malignant progression by enhancing ferroptosis resistance, providing a promising therapeutic target for overcoming ferroptosis evasion in HNSCC.

## Introduction

Head and neck squamous cell carcinomas (HNSCC) are predominantly found in the oral cavity, oropharynx, and larynx, making it the sixth most prevalent cancer globally, with over 600,000 new diagnoses annually across the world (Siegel et al., 2023). Owing to the hidden nature of their anatomical sites, many HNSCC cases are not detected until the disease has reached an advanced stage (Johnson et al., 2020). Although the management of HNSCC includes a combination of surgery, radiation, chemotherapy, targeted treatments, and immune therapies, the survival rates for patients have shown minimal progress over the last decade (Maghami et al., 2020; Gu et al., 2022). Thus, it is urgent to elucidate the molecular mechanisms underlying HNSCC pathogenesis in order to identify potential therapeutic strategies.

Ferroptosis, a recently recognized type of cell death, is driven by iron-dependent lipid peroxidation (Stockwell, 2022). Morphologically, ferroptosis is characterized by increased mitochondrial membrane density, a significant reduction in cristae, and mitochondrial shrinkage (Stockwell, 2022). Lipid peroxidation, as the central mechanism of ferroptosis, leads to the oxidative degradation of lipids, subsequently forming peroxides and hydroperoxide derivatives (Rochette et al., 2022). This reaction mainly produces lipid hydroperoxides; among its secondary metabolites, malondialdehyde (MDA) is the most mutagenic, and 4-hydroxynonenal (4-HNE) possesses the highest toxicity (Ayala et al., 2014). It has been discovered that cells possess multiple defense mechanisms against ferroptosis, primarily maintaining cell survival by counteracting lipid peroxidation (Stockwell, 2022; Dixon and Pratt, 2023). Among these, the glutathione peroxidase 4 (GPX4)/glutathione (GSH) system serves as the main defense, with solute carrier family 7 member 11 (SLC7A11) playing a crucial role as its upstream molecule (Jiang et al., 2021; Wang et al., 2023a). Emerging preclinical data suggest that inducing ferroptosis may offer a potent therapeutic option for cancer treatment (Lei et al., 2022; Wang et al., 2023b).

Triose phosphate isomerase 1 (TPI1, also known as TPI/TIM), located at 12p13.31, catalyzes the reversible interconversion of triose phosphate intermediates, which is essential for glycolysis and cellular energy homeostasis (Maquat et al., 1985; Ansari-Lari et al., 1996; Chen et al., 2017; Bejarano et al., 2021). Mounting evidence has confirmed that TPI1 is overexpressed in bladder and breast cancers, correlating with poor prognosis. It acts as an oncogenic driver to promote proliferation, migration, invasion, and gemcitabine resistance via p53 ubiquitination, autophagy activation, and glycolytic reprogramming (Jin et al., 2022; Wang et al., 2025a; Wang et al., 2025b). However, the expression pattern, clinical significance, and biological roles of TPI1 in HNSCC remain largely unexplored. Notably, whether TPI1 participates in regulating ferroptosis in HNSCC has not been reported to date.

In the present study, we comprehensively analyzed the expression pattern of TPI1 in HNSCC via TCGA database mining, clinical sample validation, single-cell RNA sequencing, and spatial transcriptomics. We further evaluated the clinical correlation and prognostic significance of TPI1 in HNSCC patients. *In vitro* functional experiments demonstrated that TPI1 promotes the proliferation, migration, and invasion of HNSCC cells. More importantly, we revealed that TPI1 enhances resistance to ferroptosis in HNSCC cells by regulating lipid peroxidation, reactive oxygen species accumulation, and intracellular Fe^2+^ levels. Collectively, our findings uncover the previously unrecognized oncogenic role of TPI1 in HNSCC progression and establish a novel functional link between TPI1 and ferroptosis regulation, providing a promising prognostic biomarker and potential therapeutic target for HNSCC.

## Materials and methods

### Data mining of TPI1 in TCGA

Gene expression data (TPM, log_2_ (value + 1)-transformed) of TPI1 across 33 cancer types, including tumor tissues and adjacent normal tissues, were obtained from The Cancer Genome Atlas (TCGA) database. All statistical analyses and data visualization were performed using R software (version 4.2.1) with the following packages: stats (version 4.2.1), car (version 3.1–0), and ggplot2 (version 3.4.4). Based on the distribution characteristics of the data, the Wilcoxon rank sum test was used to compare the expression differences between tumor and normal tissues; analyses were only performed when the statistical assumptions were satisfied. Data visualization was conducted using the ggplot2 package.

### Human tissue samples

This study was approved by the Ethics Committee of the First Affiliated Hospital of Anhui Medical University, and written informed consent was obtained from all patients. Fresh HNSCC tissues and paired adjacent normal tissues were collected and divided into two parts: one part was immediately stored at −80 °C for Western blot assays, and the remaining tissues were fixed in 10% neutral buffered formalin for subsequent immunohistochemical (IHC) staining (Supplementary Table S1).

### Clinical correlation analysis and prognostic analysis

Correlations between TPI1 expression and key clinicopathologic features, including patient sex, histologic grade, and pathologic stage, were assessed in the TCGA-HNSC cohort using the Wilcoxon rank-sum test. Patients were stratified into high- and low-TPI1 expression groups using the median expression value as the cutoff. Kaplan-Meier survival analysis with the log-rank test was performed to evaluate the association between TPI1 expression and overall survival (OS), and subgroup analyses were conducted across clinically relevant strata. Based on the results of multivariate Cox proportional hazards regression, a prognostic nomogram integrating TPI1 expression and independent clinical covariates was constructed to improve risk stratification for HNSCC patients. The predictive performance of the nomogram was validated using calibration curves and receiver operating characteristic (ROC) curve analysis. All statistical analyses were performed in R, utilizing the survival, survminer, and rms packages.

### Single-cell analysis of TPI1

To characterize the expression pattern of TPI1 in the tumor microenvironment of HNSCC, we retrieved three publicly available single-cell RNA-sequencing (scRNA-seq) datasets (GSE103322, GSE150321, and GSE172577) from the Tumor Immune Single-Cell Hub 2 (TISCH2) database (https://tisch.comp-genomics.org/). All datasets were uniformly preprocessed by the TISCH2 pipeline, including quality control, normalization, principal component analysis (PCA), Uniform Manifold Approximation and Projection (UMAP) dimensionality reduction, and cell-type annotation based on canonical lineage markers. For each dataset, the cellular composition was visualized using UMAP embeddings colored by cell-type labels, and the distribution of TPI1 expression across annotated cell populations was characterized via feature plots overlaying expression levels on the UMAP space (with color intensity representing relative gene expression) and violin plots summarizing expression levels across distinct cell types.

### Spatial transcriptomics and correlation analysis of TPI1 in the HNSCC tumor microenvironment

Spatial transcriptomics data from four HNSCC tissue sections (HNSC1–4) generated by the 10X Visium platform were analyzed. H&E-stained tissue images and Seurat objects were loaded, and raw counts were LogNormalize-normalized (scale factor = 10,000). The expression of TPI1 (raw counts and normalized levels) was visualized on tissue coordinates using Seurat SpatialFeaturePlot. Cell-type composition at each spot was inferred using Cottrazm spatial deconvolution, which integrates spatial transcriptomics, H&E images, and scRNA-seq references. Deconvolution results were visualized as dominant cell-type maps and individual cell-type proportion plots. Spearman’s rank correlation test was used to assess the spot-level association between TPI1 expression and cell-type proportions, with significant correlations visualized using linkET arc plots.

### Cell culture and transfection

FaDu cells were purchased from the American Type Culture Collection (ATCC), and TU177 cells were obtained from Otwo Biotech (Shenzhen, China). All cell lines were confirmed to be Mycoplasma-free using the MycoAlert system (Lonza, LT07-318). All cells were cultured in DMEM (Sigma, D6429) supplemented with 10% fetal bovine serum (Gibco, 10,437–028) and 1% penicillin–streptomycin (Beyotime, C0222) at 37 °C with 5% CO_2_. Lentiviruses for TPI1 knockdown and TPI1 overexpression were constructed and packaged by GeneChem (Shanghai, China). Human TPI1 shRNA sequences: #1, 5′- TGA​TGT​GGA​TGG​CTT​CCT​TGT-3′, #2, 5′- CCC​TAC​TGC​CTA​TAT​CGA​CTT-3′.

### Western blotting

Western blotting was performed as previously reported (Wang et al., 2024). Briefly, cells were lysed with RIPA buffer (B14002, Bimake) containing protease and phosphatase inhibitors (B15002, Bimake). Protein concentrations were determined using the BCA assay (Beyotime, P0010), followed by heating with loading buffer for 5 min. Proteins were separated by SDS-PAGE (10% gel) and transferred to PVDF membranes (Millipore, IPVH00010). Membranes were blocked with 5% skim milk for 1 h and incubated with primary antibodies overnight at 4 °C. After washing, secondary antibodies were applied at room temperature for 1 h, and protein signals were visualized using chemiluminescence.

### RNA extraction and quantitative real-time PCR (qRT-PCR)

Total cellular RNA was extracted using RNAiso Plus (Takara, 9,108) following the manufacturer’s instructions. The purity and concentration of the RNA were assessed using a NanoDrop 2000 spectrophotometer. One microgram of total RNA was reverse-transcribed into cDNA using the PrimeScript RT reagent kit with gDNA Eraser (RR037A, Takara). qRT-PCR was performed using TB Green Premix (RR820A, Takara) on a LightCycler® 96 system. Primer sequences for amplification were as follows: TPI1 forward primer, 5′-GCA​TCA​CTG​AGA​AGG​TTG​TTT​T-3′ and reverse primer, 5′-AGA​GCC​TCC​ATA​AAT​GAT​ACG​G-3′; GAPDH forward primer, 5′-GTA​TCG​TGG​AAG​GAC​TCA​TGA​C-3′ and reverse primer, 5′-ACC​ACC​TTC​TTG​ATG​TCA​TCA​T-3′.

### Antibodies

Primary antibodies used for Western blotting were as follows: rabbit polyclonal anti-TPI1 (10713-1-AP, 1:2000, Proteintech) and mouse monoclonal anti-β-actin (66009-1-Ig, 1:20,000, Proteintech), both obtained from Proteintech. For IHC staining, rabbit polyclonal anti-TPI1 (10713-1-AP, 1:200, Proteintech) was utilized.

### IHC

Formalin-fixed tissues were paraffin-embedded and sliced into 4 μm sections. These tissue sections were then subjected to immunostaining using the MaxVision Kit (Maixin Biol, KIT-5020) following the manufacturer’s protocol. The primary antibodies were applied as specified. A total of 50 μL of MaxVision reagent was added to each slide. The color development was achieved using 0.05% diaminobenzidine and 0.03% hydrogen peroxide in 50 mM Tris-HCl (pH 7.6), followed by counterstaining with hematoxylin. The slides were analyzed using a digital slide scanner (Aperio CS2).

### Cell viability assay

Cell viability was detected by CCK-8 assay in two independent assays. For RSL3 (HY-100218A, MCE) IC50 determination, HNSCC cells were seeded at 1 × 10^4^ cells/well, incubated with CCK-8 reagent (C0037; Beyotime) for 1 h, and absorbance at 450 nm was measured. For long-term cell survival detection, cells were plated at 1.5 × 10^3^ cells/well, and viability was detected at 24, 48, 72, 96 h using CCK-8. After incubation for 2 h at 37 °C, OD values at 450 nm were collected.

### Colony formation assay

HNSCC cells (FaDu and TU177) were seeded in 6-well plates at 500–1,000 cells per well and cultured in complete DMEM at 37 °C with 5% CO_2_. The medium was refreshed every 3 days. After 10–14 days of incubation, cells were fixed with 4% paraformaldehyde, stained with 0.1% crystal violet, rinsed with PBS, air-dried, and colonies containing more than 50 cells were counted.

### Wound healing assay

HNSCC cells (FaDu and TU177) were seeded in 6-well plates and cultured to 80% confluency. A uniform scratch was generated in the cell monolayer with a 200 μL pipette tip. After scratching, the medium was refreshed. The wound width was measured at 0 h and 24 h via ImageJ software to evaluate cell migration ability.

### Cell migration and invasion assays *in vitro*

*In vitro* migration and invasion abilities were assessed by Transwell assay (Corning, NY, United States, #3422). For invasion assay, the upper chamber was pre-coated with Matrigel (Corning, #354234), diluted 1:3 with serum-free DMEM and incubated at 37 °C for 1.5 h to form a gel. HNSCC cells (FaDu and TU177) were resuspended in serum-free DMEM at 2 × 10^4^ cells/200 μL, then added to the upper chamber; the lower chamber was filled with 600 μL complete DMEM containing 20% FBS. After 24 h of incubation at 37 °C with 5% CO_2_, non-migrated/non-invaded cells on the upper membrane were removed, and the stained cells on the lower membrane were counted under a microscope.

### Malondialdehyde (MDA) assay

HNSCC cells (FaDu and TU177) were treated with ferroptosis inducers for 12 h. Cells were harvested and lysed, and cellular MDA content was measured using a commercial MDA assay kit (S0131M, Beyotime) according to the manufacturer’s protocols. The supernatant of cell lysates was mixed with reaction reagents, incubated at 95 °C for 30 min, and the absorbance was detected at 532 nm. The MDA concentration was calculated based on the standard curve.

### Lipid peroxidation assay

The BODIPY 581/591 C^11^ staining assay (Thermo Fisher Scientific, D3861) was employed to assess lipid peroxidation. The cells were cultured in a 12-well plate at a density of 1 × 10^5^ cells per well and incubated at 37 °C with 5% CO_2_ as per the indicated protocol. For the last 30 min of incubation, 1.5 μM BODIPY 581/591 C^11^ dye was added to the cells. After two washes with Hanks’ Balanced Salt Solution (HBSS; Macgene, cc025), the cells were suspended in HBSS and analyzed using flow cytometry.

### Iron assay

HNSCC cells (FaDu and TU177) were treated with ferroptosis inducers for 12 h, then harvested and lysed. Fe^2+^ levels were measured using an Abcam iron assay kit as per the manufacturer’s instructions (ab83366). Briefly, cell lysates were centrifuged, and the supernatant was mixed with assay buffer and iron probe, incubated away from light, then absorbance at 593 nm was measured to calculate Fe^2+^ concentration via the standard curve.

### Statistical analysis

All statistical analyses were conducted using R software (version 4.2.1) and GraphPad Prism 9.5 (La Jolla, California, United States). Student’s t-test and Wilcoxon rank-sum test were applied to evaluate the statistical significance of intergroup disparities, and Spearman’s correlation analysis was implemented to quantify pairwise correlations between variables. The survival rates were calculated by the Kaplan-Meier method. Statistical significance was defined as *P < 0.05, **P < 0.01, ***P < 0.001, and ****P < 0.0001.

## Results

### TPI1 is overexpressed in HNSCC and correlates with poor patient survival

**FIGURE 1:**
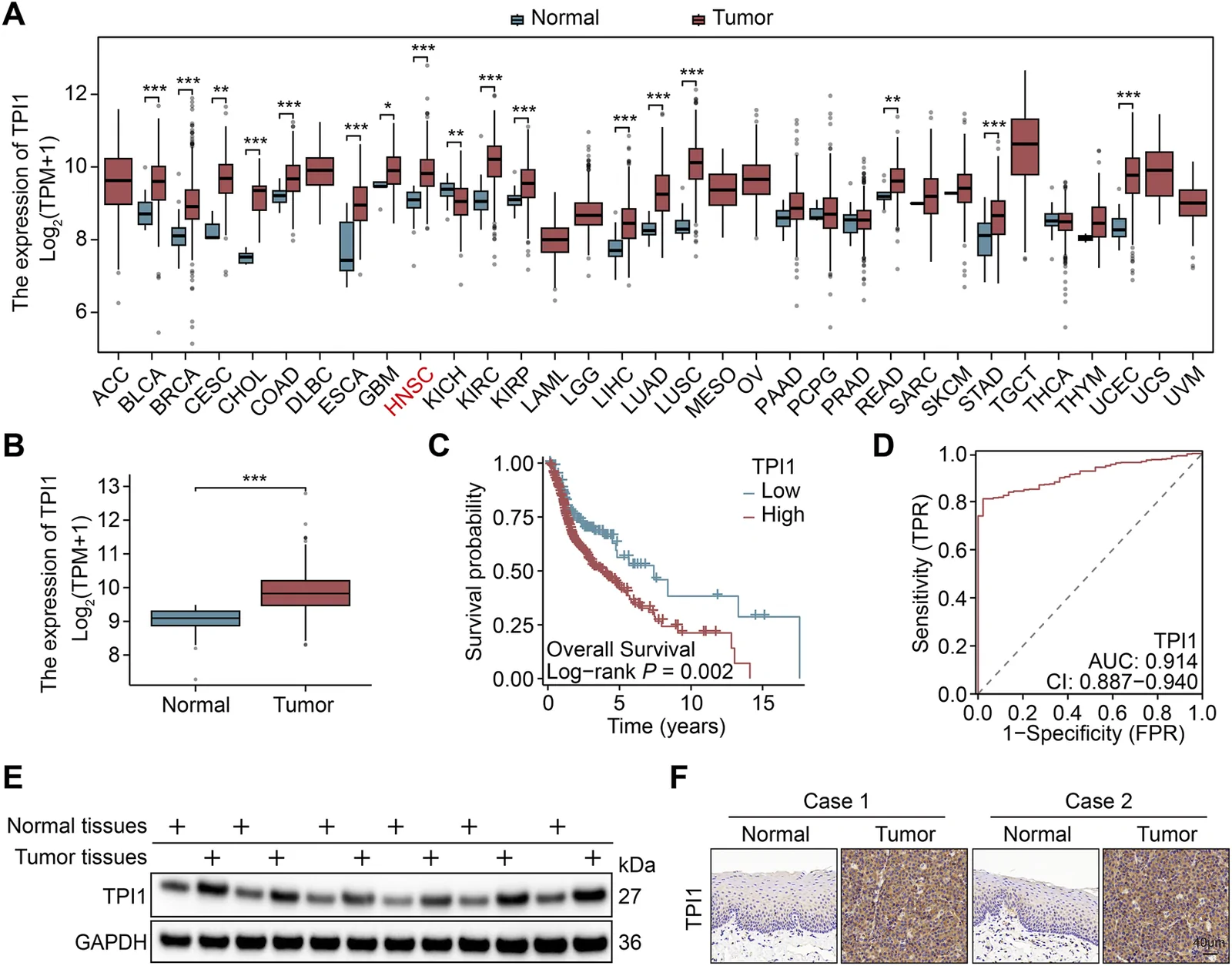
TPI1 is upregulated in HNSCC and correlates with poor prognosis. **(A)** RNA expression of TPI1 across various cancers and normal tissues from TCGA. **(B)** Expression of TPI1 in TCGA-HNSCC databases. **(C)** Kaplan-Meier survival curves for OS in high vs. low TPI1 expression groups. **(D)** Receiver operating characteristic (ROC) curve of TPI1 for distinguishing HNSCC tumors from normal tissues. **(E)** Western blot detection of TPI1 protein expression in tissues. **(F)** IHC staining to detect the expression and localization of TPI1 in HNSCC specimens. Scale = 40 μm.

We first examined the expression landscape of TPI1 across multiple human malignancies using TCGA pan-cancer data. As shown in Figure 1A, TPI1 mRNA levels were significantly elevated in the majority of tumor types compared with corresponding normal tissues, with particularly robust upregulation observed in HNSCC. A focused analysis of the TCGA-HNSCC cohort confirmed that TPI1 transcript expression was markedly higher in primary tumor tissues than in matched adjacent normal tissues (Figure 1B). To evaluate the prognostic relevance of TPI1, patients were stratified into high- and low-expression subgroups based on TPI1 mRNA levels. Kaplan-Meier survival analysis demonstrated that HNSCC patients with high TPI1 expression exhibited significantly worse overall survival compared with those with low TPI1 expression (Figure 1C). The diagnostic performance of TPI1 in distinguishing HNSCC from normal tissues was further assessed by receiver operating characteristic (ROC) curve analysis, which yielded an area under the curve (AUC) of 0.914 (95% confidence interval: 0.887–0.940), indicating high sensitivity and specificity for TPI1 as a tumor biomarker (Figure 1D).

To validate these findings at the protein level, Western blot analysis was performed on six pairs of matched HNSCC tumor and adjacent normal tissues. Consistently, TPI1 protein expression was substantially increased in tumor specimens compared with their normal counterparts (Figure 1E). Immunohistochemical staining of two representative clinical pairs further confirmed robust upregulation of TPI1 protein in HNSCC tissues relative to adjacent normal epithelium (Figure 1F). Collectively, these data demonstrate that TPI1 is frequently overexpressed in HNSCC at both the mRNA and protein levels, and that elevated TPI1 expression correlates with poor clinical outcome, supporting its potential as a prognostic biomarker for HNSCC.

### Clinical correlates and prognostic utility of TPI1 expression in HNSCC

**FIGURE 2:**
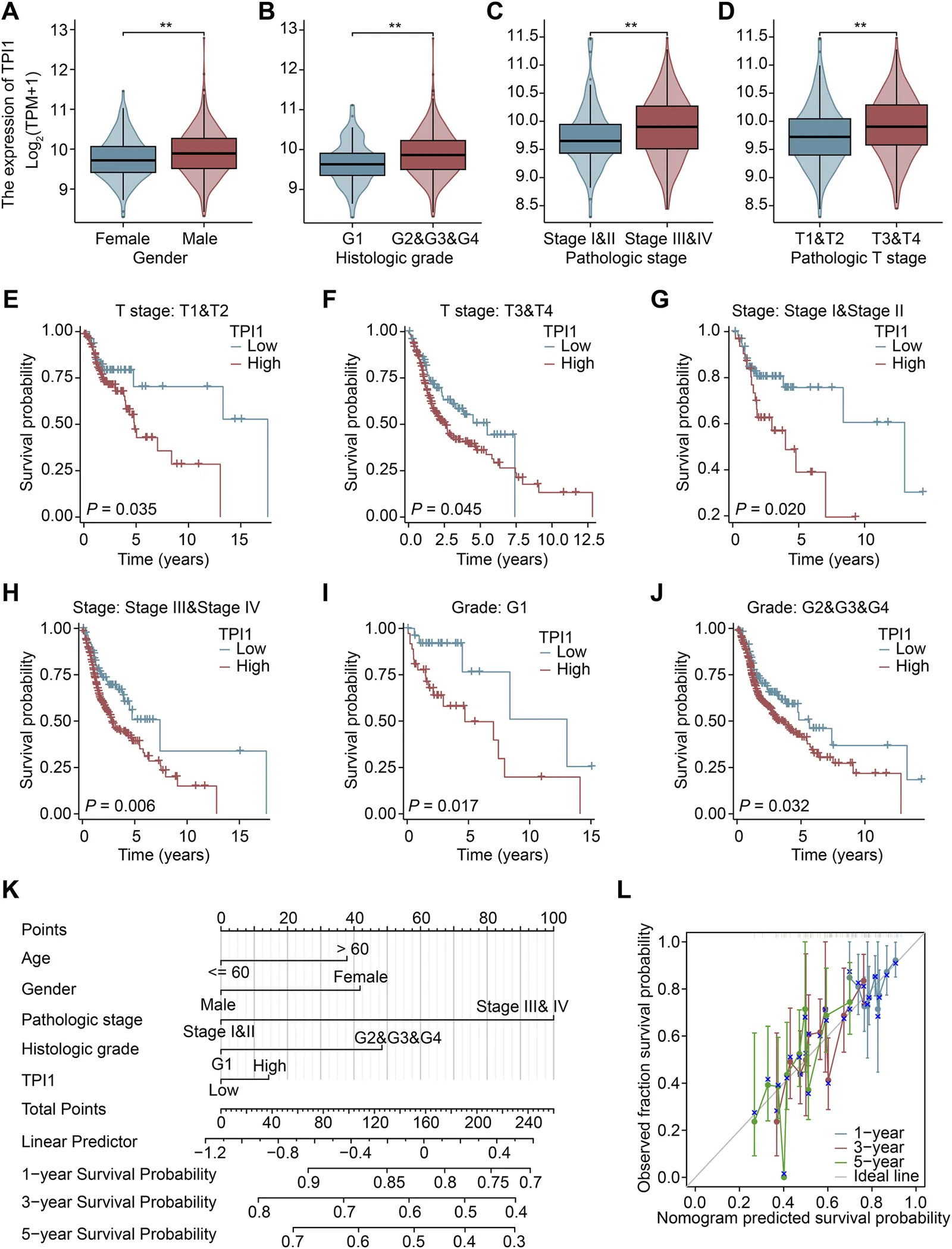
Association of TPI1 with clinical characteristics and prognostic indicators in HNSCC. **(A-D)** Expression of TPI1 across subgroups defined by gender, histologic grade, pathologic stage, and pathologic T stage in HNSCC. **(E-J)** Kaplan-Meier survival curves for OS in high vs. low TPI1 expression groups across clinical subgroups. **(K)** Nomogram based on significant indicators from multivariate COX regression for predicting OS. **(L)** Calibration curves at 1, 3, and 5 years for the OS nomogram.

To further investigate the clinical correlates of TPI1 expression in HNSCC, we analyzed its distribution across key clinicopathologic parameters in the TCGA cohort. TPI1 mRNA levels were significantly higher in male patients compared with female patients (Figure 2A). When stratified by histologic grade, TPI1 expression was significantly elevated in patients with high-grade tumors (G2–G4) relative to those with low-grade tumors (G1; Figure 2B). Similarly, patients with advanced pathologic stage (III–IV) or advanced T stage (T3–T4) exhibited significantly higher TPI1 expression than those with early-stage disease (I–II) or early T stage (T1–T2; Figures 2C,D).

We next performed subgroup survival analyses to assess whether the prognostic impact of TPI1 expression was consistent across different disease stages. In patients with early T stage (T1–T2), high TPI1 expression was associated with significantly worse overall survival (Figure 2E), a trend that remained significant in patients with advanced T stage (T3–T4; Figure 2F). Consistent with these findings, high TPI1 expression predicted poorer survival in both early-stage (I–II; Figure 2G) and advanced-stage (III–IV; Figure 2H) HNSCC patients. Similarly, stratification by histologic grade revealed that high TPI1 expression correlated with significantly reduced survival in both low-grade (G1; Figure 2I) and high-grade (G2–G4; Figure 2J) subgroups.

To integrate TPI1 expression with key clinical variables for prognostic risk stratification, we constructed a nomogram incorporating age, gender, pathologic stage, histologic grade, and TPI1 expression level (Figure 2K). Each variable was assigned a weighted point value, with the total score corresponding to predicted 1-, 3-, and 5-year survival probabilities. Calibration curve analysis demonstrated strong agreement between the nomogram-predicted survival probabilities and the actual observed survival rates, indicating favorable predictive accuracy (Figure 2L). Collectively, these results demonstrate that TPI1 expression correlates with aggressive clinicopathologic features and serves as a consistent prognostic marker across diverse patient subgroups in HNSCC.

### TPI1 is highly expressed in malignant cells across HNSCC single-cell datasets

To pinpoint the cellular origin of TPI1 upregulation in HNSCC, we performed an integrative analysis of three independent, publicly available single-cell RNA-seq datasets: GSE103322, GSE150321, and GSE172577. These datasets collectively include transcriptomic profiles of thousands of cells derived from primary HNSCC tumors, allowing us to map TPI1 expression at single-cell resolution within the heterogeneous tumor microenvironment.

**FIGURE 3:**
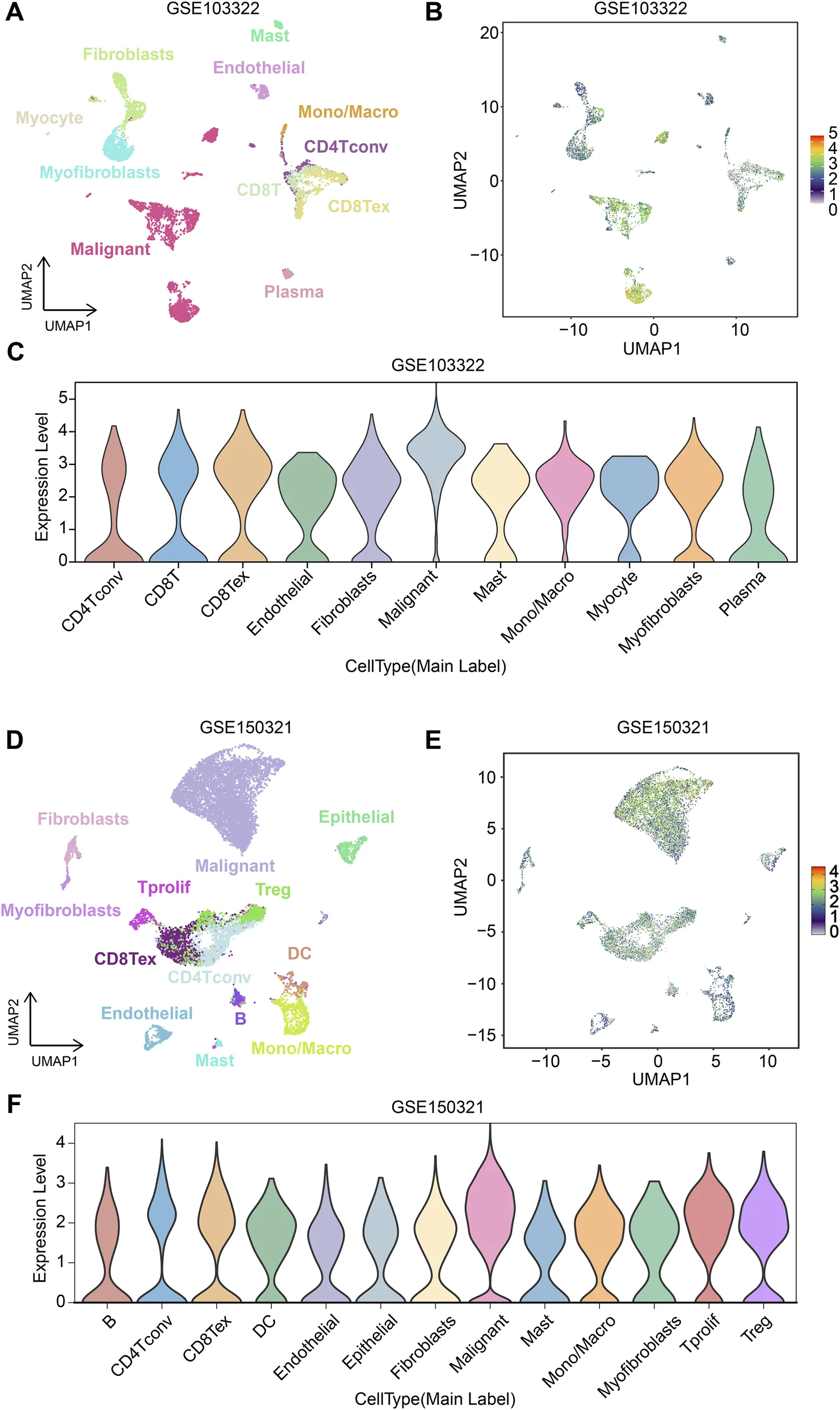
Single-cell transcriptomic profiling of TPI1 expression in HNSCC. **(A,D)** UMAP plots showing cell type annotation of GSE103322 **(A)** and GSE150321 **(D)** datasets. **(B,E)** Feature plots displaying TPI1 expression across cells in GSE103322 **(B)** and GSE150321 **(E)**. **(C,F)** Violin plots depicting the distribution of TPI1 expression across different cell types in GSE103322 **(C)** and GSE150321 **(F)**.

First, we conducted unsupervised graph-based clustering and visualized the resulting cell populations using Uniform Manifold Approximation and Projection (UMAP). This analysis identified all major cellular compartments in each dataset, including malignant epithelial cells, stromal cells (cancer-associated fibroblasts, endothelial cells), and a diverse repertoire of immune cells (e.g., CD4+/CD8+ T cells, monocytes/macrophages, dendritic cells, NK cells, and mast cells) (Figures 3A,D; Supplementary Figure S1A). To assess the distribution of TPI1 expression across these populations, we generated feature projection plots, which revealed a striking pattern: in all three cohorts, TPI1 expression was most prominent and widely detected within the malignant cell cluster (Figures 3B,E; Supplementary Figure S1B). By contrast, TPI1 expression in stromal and immune cells was generally lower and more heterogeneous across datasets.

Quantitative analysis using violin plots further confirmed this observation. Across all three datasets, TPI1 transcript levels were significantly higher in malignant cells than in any other cell type, including epithelial cells, fibroblasts, endothelial cells, and all immune cell subsets (Figures 3C,F; Supplementary Figure S1C). This pattern was consistent regardless of the specific dataset or cell annotation scheme used, indicating that TPI1 enrichment in malignant cells is a robust and generalizable feature of HNSCC. Collectively, these single-cell data demonstrate that the elevated TPI1 expression observed in bulk tumor tissues is not driven by stromal or immune components, but rather is predominantly contributed by the malignant epithelial cell compartment in HNSCC.

### Spatial mapping of TPI1 in the HNSCC tumor microenvironment

**FIGURE 4:**
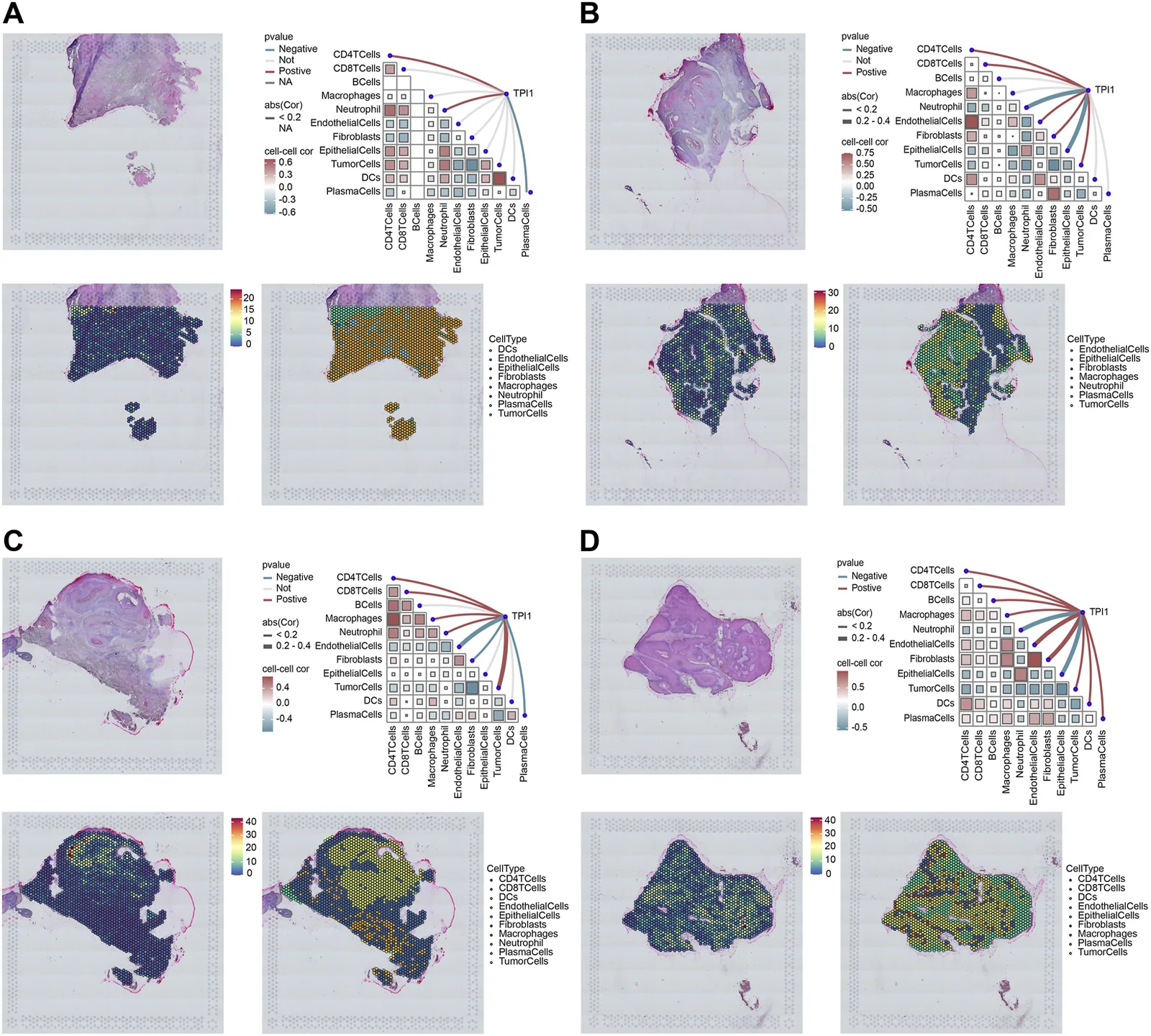
Spatial transcriptomic profiling of TPI1 in the HNSCC tumor microenvironment. **(A–D)** Representative spatial transcriptomic profiles of four HNSCC patient samples (HNSC1–HNSC4). For each sample: the top left panel shows the H&E-stained tissue section; the top right panel displays the correlation matrix and interaction network between TPI1 expression and major cell types; the bottom left panel presents the spatial distribution of TPI1 expression (color gradient); the bottom right panel illustrates the spatial localization of annotated cell types in the tumor microenvironment.

To investigate the spatial distribution and cellular context of TPI1 expression in the HNSCC tumor microenvironment, we analyzed publicly available spatial transcriptomic datasets containing four independent patient samples. First, we examined hematoxylin and eosin (H&E) staining images and the corresponding spatial expression maps of TPI1 across all samples (Figures 4A–D). TPI1 expression was predominantly localized within morphologically defined tumor regions, showing clear enrichment in the malignant tissue compartment. Cell-type deconvolution further confirmed that regions with high TPI1 expression strongly overlapped with tumor cell-enriched areas, reinforcing the malignant cell-specific expression pattern of TPI1 at spatial resolution. To explore the relationship between TPI1 and the tumor microenvironment, we performed cell-cell correlation analysis. In all four samples, TPI1 expression exhibited the strongest positive correlation with tumor cells. In addition, TPI1 showed significant positive correlations with several immune cell populations, including macrophages and dendritic cells, suggesting potential crosstalk between TPI1-expressing tumor cells and the immune microenvironment (Figures 4A–D). Collectively, these spatial transcriptomic analyses from public datasets demonstrate that TPI1 is primarily expressed in the malignant cell compartment *in situ* and correlates with specific immune cell subsets. These findings point to potential crosstalk between TPI1-expressing tumor cells and the immune microenvironment, while the underlying causal link awaits further experimental verification.

### Knockdown of TPI1 suppresses proliferation, migration, and invasion of HNSCC cells *in vitro*

**FIGURE 5:**
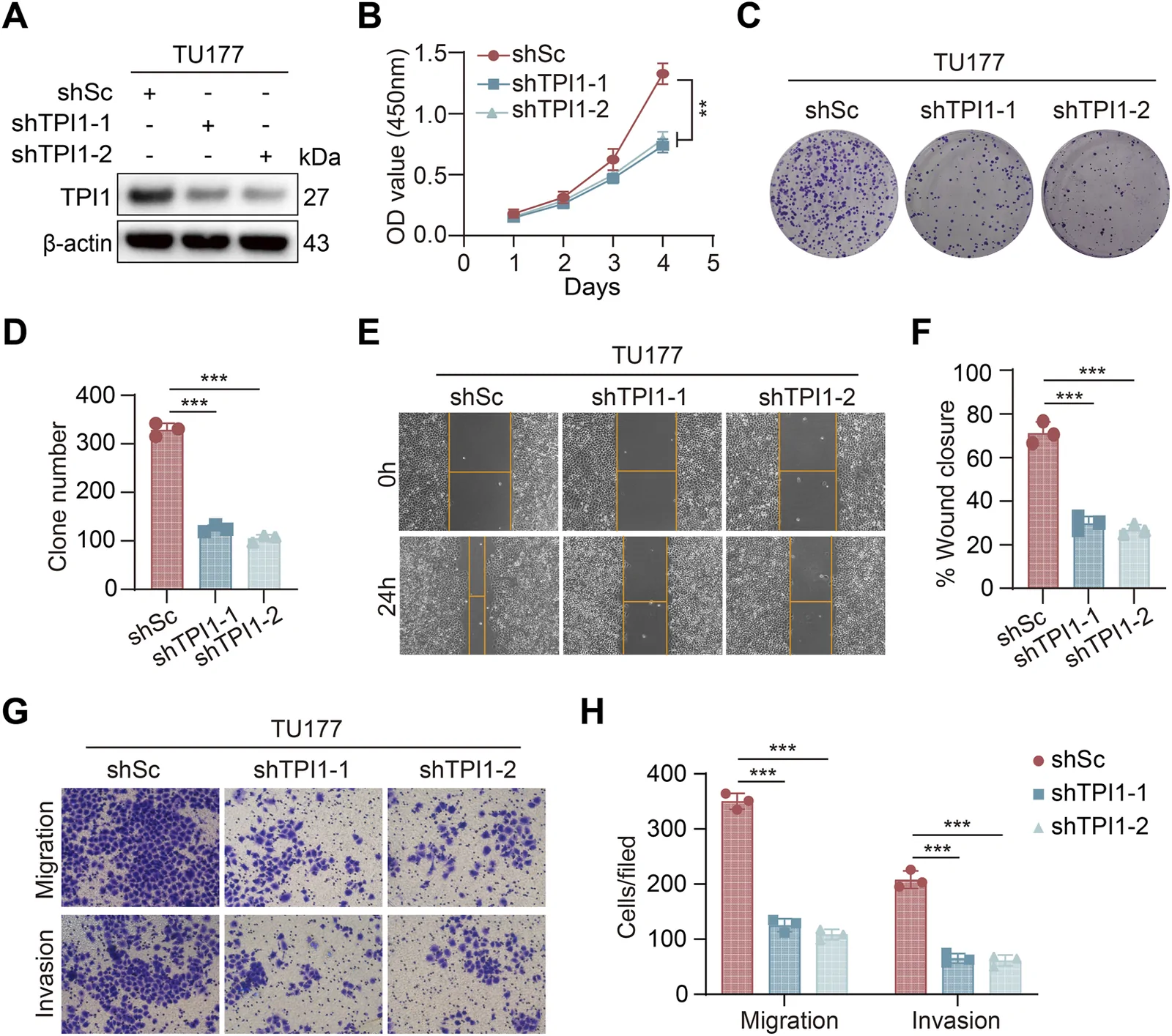
TPI1 knockdown inhibits proliferation, colony formation, migration, and invasion of TU177 HNSCC cells. **(A)** Western blot showing TPI1 protein levels in TU177 cells transfected with control shRNA (shSc) or two independent shRNAs targeting TPI1. **(B)** CCK-8 assay measuring cell proliferation over 4 days. **(C,D)** Representative images **(C)** and quantification **(D)** of colony formation assays. **(E,F)** Representative images **(E)** and quantification **(F)** of wound-healing assays at 0 h and 24 h to assess cell migration. **(G,H)** Representative images **(G)** and quantification **(H)** of transwell migration and invasion assays.

To investigate the functional role of TPI1 in HNSCC progression, we performed loss-of-function experiments in the TU177 cell line using two independent short hairpin RNAs (shRNAs) targeting TPI1 (shTPI1-1 and shTPI1-2). Western blotting and qRT-PCR confirmed efficient knockdown of TPI1 at protein and mRNA levels in both shTPI1 groups compared with the non-targeting shSc control (Figure 5A; Supplementary Figure S2A). Cell proliferation assays showed that TPI1 knockdown significantly inhibited the growth of TU177 cells over a 4-day culture period (Figure 5B). Consistently, colony formation assays revealed a marked reduction in colony number in both shTPI1 groups, indicating impaired long-term proliferative capacity (Figures 5C,D). We next assessed the effect of TPI1 knockdown on cell migration and invasion. Wound healing assays demonstrated that the closure rate of the scratch wound was significantly lower in TPI1-knockdown cells than in control cells, suggesting reduced migratory ability (Figures 5E,F). Furthermore, Transwell migration and invasion assays confirmed that both the migratory and invasive capacities of TU177 cells were substantially attenuated following TPI1 silencing (Figures 5G,H). Collectively, these *in vitro* functional assays indicate that TPI1 plays a critical role in promoting the proliferation, migration, and invasion of HNSCC cells.

### Overexpression of TPI1 promotes proliferation, migration, and invasion of HNSCC cells *in vitro*

**FIGURE 6:**
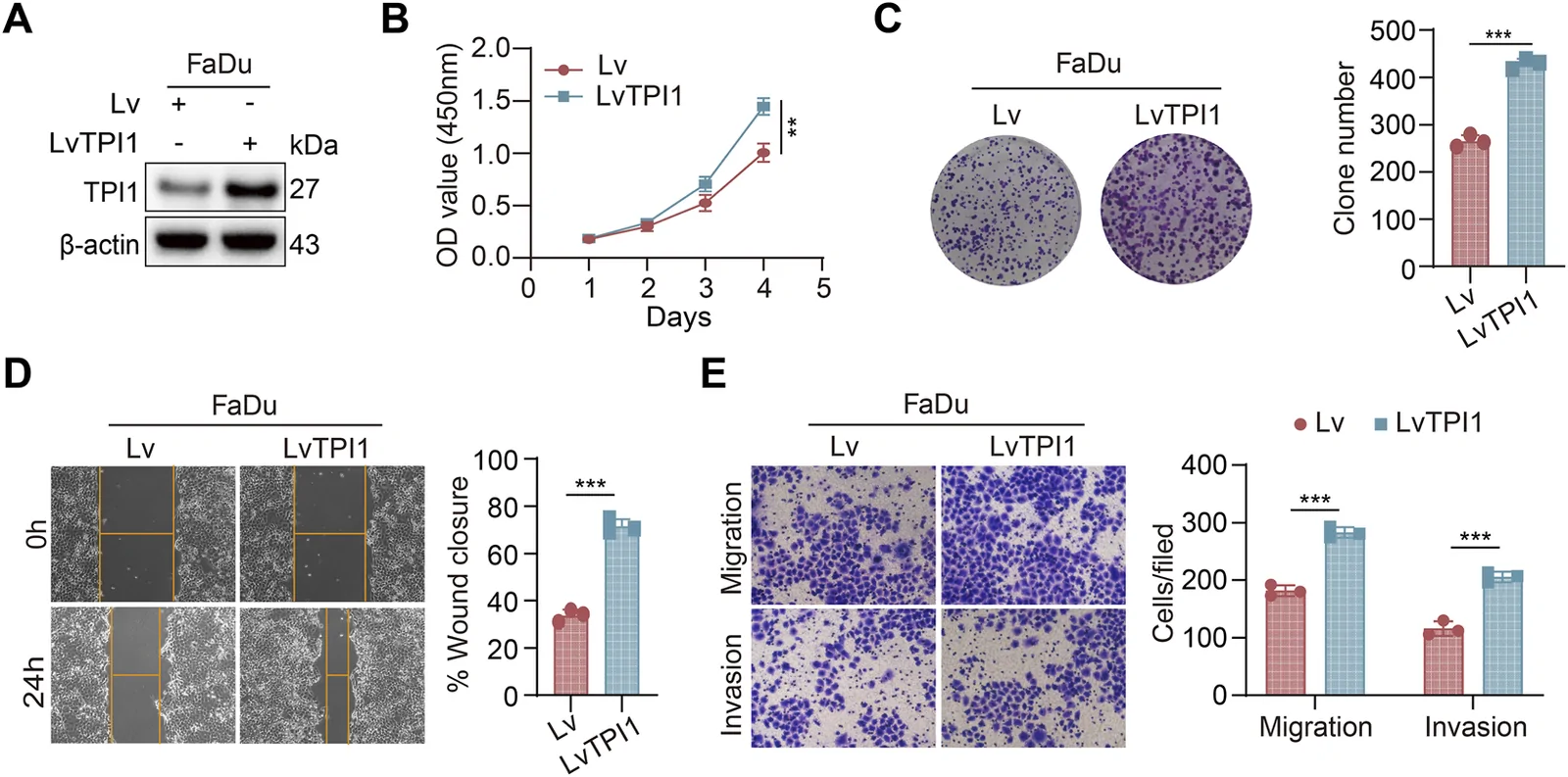
Overexpression of TPI1 promotes proliferation, colony formation, migration, and invasion in FaDu HNSCC cells. **(A)** Western blot showing TPI1 protein levels in FaDu cells transduced with control lentivirus (Lv) or TPI1-overexpressing lentivirus (LvTPI1). **(B)** CCK-8 assay measuring cell proliferation over 4 days. **(C)** Representative images and quantification of colony formation assays. **(D)** Representative bright-field images of wound-healing assays captured at 0 h and 24 h, with corresponding quantitative analysis of wound closure rate. **(E)** Representative staining images and quantitative cell counting of transwell migration and invasion assays.

To further confirm the functional role of TPI1 in HNSCC progression, we performed gain-of-function experiments in the FaDu cell line using lentivirus-mediated overexpression of TPI1 (LvTPI1). Western blotting and qRT-PCR verified that TPI1 mRNA and protein expression were significantly upregulated in the LvTPI1 group compared with the empty vector control (Lv) (Figure 6A; Supplementary Figure S2B). Cell proliferation assays showed that TPI1 overexpression significantly enhanced the growth of FaDu cells over a 4-day culture period (Figure 6B). Consistently, colony formation assays revealed a marked increase in colony number in the LvTPI1 group, indicating enhanced long-term proliferative capacity (Figure 6C). Next, we assessed the effect of TPI1 overexpression on cell migration and invasion. Wound healing assays demonstrated that the closure rate of the scratch wound was significantly higher in TPI1-overexpressing cells than in control cells, suggesting enhanced migratory ability (Figure 6D). Furthermore, Transwell migration and invasion assays confirmed that both the migratory and invasive capacities of FaDu cells were substantially increased following TPI1 overexpression (Figure 6E). Collectively, these gain-of-function experiments mirror the results of the previous knockdown studies, demonstrating that TPI1 plays a critical oncogenic role in promoting the proliferation, migration, and invasion of HNSCC cells.

### TPI1 confers resistance to ferroptosis in HNSCC cells

To this point, our multi-omic analyses have demonstrated that TPI1 is predominantly expressed in the malignant cell compartment of HNSCC and functionally drives key oncogenic phenotypes, including enhanced proliferation, migration, and invasion. As a central metabolic enzyme in the glycolytic pathway, TPI1 is critically involved in cellular redox balance and energy metabolism, processes known to strongly influence ferroptosis susceptibility. Given the emerging evidence linking glycolytic reprogramming to ferroptosis regulation in cancer, we next sought to determine whether TPI1 also plays a functional role in modulating ferroptosis sensitivity in HNSCC cells.

**FIGURE 7:**
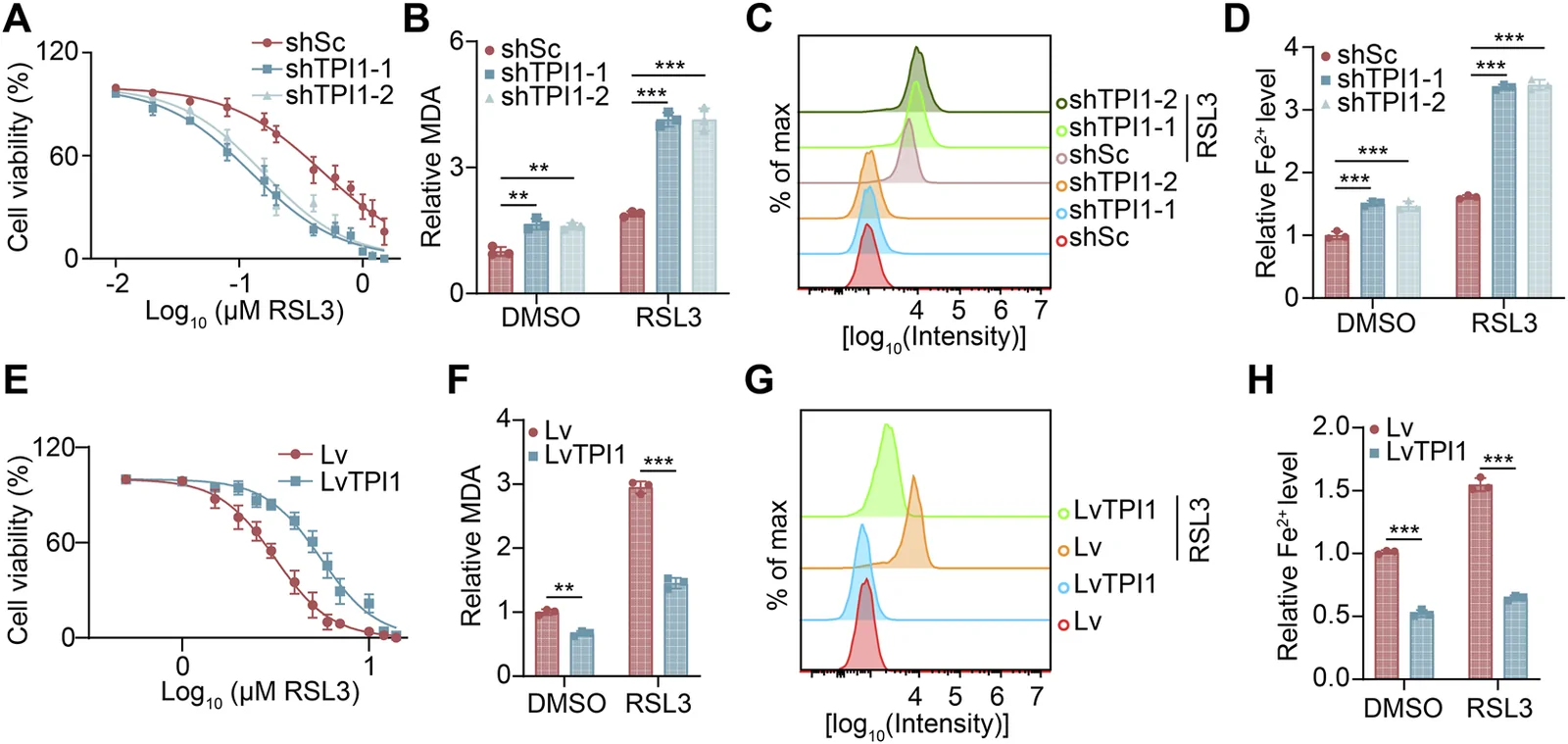
TPI1 regulates ferroptosis sensitivity in HNSCC cells. **(A,E)** CCK-8 assay–based dose–response curves of TU177 **(A)** and FaDu **(E)** cells with TPI1 knockdown (shTPI1-1/2) or overexpression (LvTPI1) treated with different RSL3 concentrations. **(B,F)** MDA levels in TU177 **(B)** and FaDu **(F)** cells treated with DMSO or RSL3. **(C,G)** Flow cytometry histograms showing lipid ROS (measured by C11-BODIPY) levels in TU177 **(C)** and FaDu **(G)** cells with TPI1 knockdown or overexpression, respectively, after RSL3 treatment. **(D,H)** Relative intracellular Fe^2+^ levels in TPI1-knockdown TU177 **(D)** and TPI1-overexpressing FaDu **(H)** cells treated with DMSO or RSL3.

We first examined the impact of TPI1 knockdown on ferroptosis induced by RSL3 in TU177 cells. Dose-response cell viability assays revealed that silencing TPI1 significantly increased cellular sensitivity to RSL3 treatment compared with the shSc control (Figure 7A). To confirm that this enhanced cell death was indeed ferroptosis-dependent, we measured key biochemical markers of ferroptosis. Upon RSL3 exposure, TPI1-knockdown cells exhibited markedly elevated levels of lipid peroxidation, as reflected by increased MDA concentration (Figure 7B). Consistently, flow cytometry analysis showed enhanced ROS accumulation (Figure 7C), and intracellular Fe^2+^ levels were also significantly increased in the TPI1-silenced group (Figure 7D). These data collectively indicate that loss of TPI1 renders HNSCC cells more susceptible to ferroptosis. Conversely, we observed the opposite phenotype in FaDu cells engineered to stably overexpress TPI1. Ectopic expression of TPI1 conferred substantial resistance to RSL3-induced cell death, as evidenced by the rightward shift in the viability dose-response curve (Figure 7E). Furthermore, while RSL3 treatment robustly induced MDA, ROS, and Fe^2+^ accumulation in control cells, these ferroptosis-associated markers were significantly attenuated in TPI1-overexpressing cells (Figures 7F–H). These results, mirroring our knockdown findings, demonstrate that TPI1 overexpression protects HNSCC cells from ferroptotic cell death.

Taken together, these complementary loss- and gain-of-function experiments establish that TPI1 plays a critical role in protecting HNSCC cells from ferroptosis. These findings reveal a previously unrecognized functional link between TPI1, a key glycolytic enzyme, and ferroptosis resistance, suggesting that TPI1 may promote tumor progression not only by driving proliferation and invasion but also by conferring metabolic protection against ferroptosis.

## Discussion

Ferroptosis is lipid peroxidation-dependent cell death that dominates therapeutic response and malignant progression in HNSCC. Multiple canonical ferroptosis effector proteins control cellular redox balance, including the anti-ferroptosis pair SLC7A11-GPX4 and pro-ferroptosis marker ACSL4. Chromatin remodeler CRB2 transcriptionally upregulates ACSL4 and LOX while repressing SLC7A11 to trigger ferroptosis in HNSCC (Zhang et al., 2026). The master antioxidant transcription factor NRF2 activates SLC7A11 and GPX4 to resist lipid peroxidation (Zhang et al., 2023); TP53 antagonizes NRF2 nuclear translocation to restore ferroptosis sensitivity (Yu et al., 2025). Although these well-established ferroptosis regulators have been widely characterized, few glycolytic enzymes have been reported to modulate ferroptosis in HNSCC.

In this study, by integrating TCGA expression data, clinical tissue validation, single-cell RNA sequencing, and spatial transcriptomics, we systematically delineated the role of TPI1 in HNSCC. TPI1 was markedly upregulated in tumor tissues, with high expression correlating with male sex, advanced stage, and poor overall survival, and serving as an independent prognostic indicator. Single-cell and spatial analyses confirmed that TPI1 is predominantly enriched in malignant epithelial cells, with minimal expression in stromal and immune compartments, highlighting its tumor-intrinsic nature. Functional assays demonstrated that TPI1 promotes HNSCC cell proliferation, migration, and invasion, while concurrently conferring resistance to ferroptosis by attenuating lipid peroxidation, ROS accumulation, and intracellular Fe^2+^ levels; conversely, TPI1 knockdown sensitized cells to ferroptotic death. Collectively, these findings indicate that TPI1 drives malignant progression and ferroptosis evasion, supporting its potential as a prognostic biomarker and therapeutic target in HNSCC.

TPI1 exhibits functional heterogeneity across different cancer types, with its pro- or anti-tumorigenic activity influenced by tissue-specific microenvironments and genetic backgrounds. In breast cancer, TPI1 promotes tumor invasion and metastasis by stabilizing CDCA5 and activating the PI3K/AKT/mTOR signaling pathway, which is highly consistent with our observation that TPI1 enhances invasive and metastatic phenotypes in HNSCC (Jin et al., 2022). In pancreatic cancer, TPI1 has been identified as a key therapeutic target for TP53-mutant subtypes, and the high prevalence of TP53 mutations in HNSCC suggests a potential crosstalk between TPI1 and common oncogenic drivers (Toyoda et al., 2024). An allelic variant of TPI1 with altered enzymatic activity has been characterized in prostate cancer cells, indicating that post-translational modifications or structural variations may further modulate TPI1 function in HNSCC (Guzmán-Luna et al., 2015). Autoantibodies against TPI1 detected in the sera of breast cancer patients support its clinical relevance and immunogenicity as a tumor-associated antigen (Tamesa et al., 2009). Notably, TPI1 acts as a tumor suppressor in hepatocellular carcinoma by inhibiting proliferation and migration, while in gastric cancer, it functions as a mediator of drug resistance reversal, highlighting the context-dependent nature of its biological role (Wang et al., 2008; Jiang et al., 2017). Naturally occurring deamidated TPI1 has also been proposed as a cell-selective therapeutic target, offering new avenues for targeted therapy (Enríquez-Flores et al., 2022). Using single-cell RNA sequencing and spatial transcriptomics, we demonstrate that TPI1’s high expression in HNSCC is tumor cell-intrinsic rather than derived from stromal or immune cells. This tumor-specific expression distinguishes HNSCC from other cancer types and reinforces its value as a precise therapeutic target. Collectively, these findings indicate that TPI1’s diverse functions across malignancies are context-dependent, and in HNSCC, its high expression contributes to tumor aggressiveness and provides a strong rationale for prognostic and therapeutic intervention.

Beyond differential expression and metabolic reprogramming, recent studies have highlighted novel mechanisms by which TPI1 modulates ferroptosis through structural and post-translational regulation. Environmental stress such as cadmium exposure can induce aggregation of TPI1, leading to loss of its catalytic function, disrupted glycolytic flux, elevated lipid peroxidation, and ferroptotic cell death, which contributes to inflammation and tissue fibrosis in the lung (Mo et al., 2024). This mechanism demonstrates that protein conformational instability of TPI1 itself can act as a direct trigger of ferroptosis, independent of transcriptional or translational changes. In contrast, at the level of post-translational modification, dopaminylation of endothelial TPI1 at the Q65 residue enhances its enzymatic activity to channel glycolytic intermediates away from pro-ferroptotic lipid synthesis and toward glucose metabolism, thereby suppressing ferroptotic signaling and promoting regenerative angiocrine function over fibrosis in lung endothelium (Yin et al., 2026). This finding not only reveals a previously unrecognized anti-ferroptotic mechanism but also illustrates that modification-dependent TPI1 activity can reversibly shift ferroptosis susceptibility in a context-specific manner. Importantly, these context-dependent mechanisms align with our observations in cisplatin-resistant oral carcinoma, where inhibition of TPI1 sensitizes tumor cells to ferroptosis, increasing ROS, free iron levels, lipid peroxidation, and ferroptotic cell death, and thereby suppressing tumor growth and chemoresistance (Wang et al., 2025c). Taken together, these studies suggest that TPI1 functions as a central nodal regulator of ferroptosis by integrating metabolic flux, structural stability, and post-translational cues to determine cellular susceptibility. Future work should explore whether similar conformational or modification-dependent regulatory mechanisms of TPI1 operate within the tumor microenvironment and how they intersect with canonical ferroptosis mediators such as GPX4, SLC7A11, and FSP1 to govern tumor progression and therapeutic response.

Building on our findings that TPI1 is highly expressed in malignant epithelial cells and contributes to ferroptosis resistance in HNSCC, we explored the association between TPI1 expression and features of the tumor immune microenvironment. Prior studies have demonstrated that TPI1 overexpression in laryngeal squamous cell carcinoma, hepatocellular carcinoma, and lung adenocarcinoma is associated with immunosuppressive features and altered immune cell infiltration patterns (Li et al., 2023; Liang et al., 2024; Liu et al., 2026). In particular, enrichment of TPI1+ tumor cells correlates with M2 macrophage polarization and a suppressive microenvironment in HCC (Liang et al., 2024; Liu et al., 2026), suggesting that TPI1 may influence macrophage phenotypes and local immune suppression via metabolic or secretory cues. Consistently, our spatial transcriptomics data in HNSCC revealed that regions with high TPI1 expression are positively associated with tumor-associated macrophages (TAMs), regulatory T cells (Tregs), and other immunosuppressive cell signatures, while inversely correlated with CD8^+^ T cell infiltration. This spatial correlation indicates a potential link between TPI1 and an immunosuppressive tumor niche, but causal inference requires further functional validation. Mechanistically, TPI1 may indirectly modulate immune cells through glycolytic metabolites and ferroptosis signaling. For instance, high TPI1 activity enhances lactate production, which has been shown to promote M2 macrophage polarization and suppress effector T cell function (Liu et al., 2026). Furthermore, TPI1-expressing tumor regions may attenuate ferroptotic signaling release, reducing pro-inflammatory immune stimulation and further reinforcing an immunosuppressive microenvironment. This multi-layered regulation of metabolism, ferroptosis, and immune interactions not only provides mechanistic insight into TPI1-mediated tumor growth, migration, and chemoresistance but also highlights TPI1 as a potential target for modulating the immune microenvironment.

Although this study systematically elucidates the role of TPI1 in regulating tumor proliferation, ferroptosis resistance, and the immune microenvironment in HNSCC, several limitations should be noted. First, the single-cell and spatial transcriptomic datasets utilized in this study were obtained from publicly available sources and included a limited number of samples; future studies incorporating additional clinical cohorts are warranted to enhance the generalizability of these findings. Second, our functional experiments were conducted exclusively in cell lines, and the role of TPI1 has not yet been validated *in vivo* models; subsequent investigations using mouse models could further clarify its impact on tumor growth and immune regulation. Third, potential heterogeneity of TPI1 function across molecular subtypes or immune contexts has not been systematically evaluated, which will be important for guiding personalized therapeutic strategies. Fourth, our conclusion that TPI1 regulates ferroptosis is only supported by cellular phenotypic data, and the precise underlying molecular mechanism remains to be further elucidated.

## Conclusion

Overall, TPI1 is highly expressed in HNSCC, promoting proliferation, migration, and invasion while suppressing ferroptosis, highlighting it as a key tumor-promoting factor and potential therapeutic target.

## Data Availability

The original contributions presented in the study are publicly available. These data can be found in the NCBI Gene Expression Omnibus (GEO) repository under the accession numbers GSE103322, GSE150321, GSE172577.

## Supplementary material

SUPPLEMENTARY FIGURE S1 Single-cell transcriptomic analysis of TPI1 expression in the GSE172577 HNSCC dataset. **(A)** UMAP plot of cell type annotations. **(B)** Feature plot showing TPI1 expression distribution. **(C)** Violin plot comparing TPI1 expression across cell types.

SUPPLEMENTARY FIGURE S2 qRT-PCR verification of TPI1 knockdown and overexpression efficiency in HNSCC cells. **(A)** Relative TPI1 mRNA levels were measured by qRT-PCR in cells transfected with negative control shSc, shTPI1-1 and shTPI1-2. **(B)** qRT-PCR analysis of TPI1 mRNA expression in cells infected with empty lentiviral vector (Lv) and TPI1 overexpression lentivirus (LvTPI1).
